# The Impact of Connective Tissue Diseases on the Inpatient Outcomes of Congestive Heart Failure Patients

**DOI:** 10.7759/cureus.11659

**Published:** 2020-11-23

**Authors:** Anantha Sriharsha Madgula, Daniel Condit, Jinjian Mu, Kai Chen

**Affiliations:** 1 Internal Medicine, University of Connecticut School of Medicine, Farmington, USA; 2 Statistics, University of Connecticut, Storrs, USA; 3 Cardiology, University of Connecticut Health Center, Farmington, USA

**Keywords:** congestive heart failure, rheumatoid arthritis, systemic lupus erythematosus, outcomes

## Abstract

Background

Rheumatoid arthritis (RA) and systemic lupus erythematosus (SLE) are autoimmune diseases with chronically elevated inflammatory activity. Treatments typically have been aimed at decreasing inflammation. While RA and SLE are known to have a high incidence of congestive heart failure (HF), the mechanism behind this remains elusive. We sought to assess the outcomes of HF patients with either RA or SLE as opposed to HF patients without RA or SLE.

Methods

We conducted a retrospective analysis of the Healthcare Utilization Project - National Inpatient Sample Database from 2010 to 2015 (third quarter). Patients with a primary admitting diagnosis of HF were queried, and those with or without a diagnosis of either SLE or RA were separated into two groups. In-hospital mortality, total charges (TOTCHG), and length of stay (LOS) were analyzed with a multivariate regression model adjusted for demographical and comorbidity variables, using generalized linear models with family binomial, gamma, and negative-binomial, respectively. A p-value smaller than 0.05 was deemed statistically significant. All the statistical analyses were performed in R 3.5.5 (R Core Team, 2013, http://www.R-project.org/).

Results

The in-hospital mortality (3.4% v/s 4.43%), mean TOTCHG ($46k v/s $51k), and mean LOS (5.79 v/s 6.12 days) were significantly lower in HF patients with RA/SLE when compared with HF patients without RA/SLE. A younger age (70.5 v/s 72.6 years) and a female preponderance (75% v/s 51%) were evident in the RA/SLE group. Both groups consistently showed a significant disparity in the rates of hospitalization, which was inversely related to household income. p-value was less than 0.001 for all the above outcomes.

Conclusions

RA/SLE patients are associated with better in-hospital outcomes of HF. The underlying mechanism is unclear in terms of this paradox. Given the fact that the majority of RA/SLE patients are treated with agents aimed at decreasing inflammation, this may shed light on the role of inflammation being an important contributor to HF and implicate a future therapeutic direction.

## Introduction

Connective tissue diseases (CTD), including rheumatoid arthritis (RA), systemic lupus erythematosus (SLE), polymyositis, and dermatomyositis, are autoimmune inflammatory disorders that can lead to multiorgan involvement. While musculoskeletal and dermatologic manifestations are traditionally at the forefront, all these disorders can lead to a range of cardiac conditions, including coronary artery disease, conduction abnormalities, valvular disorders, and myocardial and pericardial disease. A cardiac manifestation of interest is congestive heart failure (HF). Patients with CTDs have been shown to be at a higher risk of this significant cardiac comorbidity, including HF in multiple studies [1-3]. Despite the increased prevalence of HF in these patients, there are a paucity of data on the outcomes of patients with CTDs when they are hospitalized for HF.

## Materials and methods

Data source

Data were queried using the National Inpatient Sample developed in part by the Healthcare Cost and Utilization Project. It is an extensive publicly available all-payer database in the United States that represents a sample of hospitalization administrative data with approximately 35-million hospital admissions per year. Each unit of analysis is one hospital discharge, and hence readmissions are not identifiable. The database covers about 95% of the United States population and about 94% of all community hospital discharges [4].

We queried adults over the age of 18 years using the Clinical Classification Software (CCS) for the International Classification of Diseases (ICD), 9th revision, clinical modification codes that were developed as part of this database. It is a diagnosis and procedure categorization scheme that clusters multiple ICD codes into a smaller number of clinically meaningful categories.

Study population

We queried the database from January 1, 2010, to September 30, 2015, for individuals carrying a diagnosis of congestive heart failure using the CCS code 108 (Table 1). We then split this population between those carrying a diagnosis code of RA and related disorders, SLE and CTD (CCS codes 202 or 210, respectively), and those without either condition (Table 1).

**Table 1:** International Classification of Diseases-9 Code accompanying Clinical Classification Software Code 108 (congestive heart failure), 202 (rheumatoid arthritis and related disorders), 210 (systemic lupus erythematosus and connective tissue disorders) CCS = Clinical Classification Software; Dx = Diagnosis; ICD = for International Classification of Diseases

CCS Code 108			
ICD-9	Dx	ICD-9	Dx
39891	Rheumatic congestive heart failure	42831	Acute diastolic heart failure
4280	Heart failure	42832	Chronic diastolic heart failure
4281	Left heart failure	42833	Acute on chronic diastolic heart failure
42820	Systolic heart failure	42840	Combined systolic and diastolic heart failure
42821	Acute systolic heart failure	42841	Acute combined systolic and diastolic heart failure
42822	Chronic systolic heart failure	42842	Chronic combined systolic and diastolic heart failure
42823	Acute on chronic systolic heart failure	42843	Acute on chronic combined systolic and diastolic heart failure
42830	Diastolic heart failure	4289	Heart failure, unspecified
CCS Code 202			
ICD-9	Dx	ICD-9	Dx
7140	Rheumatoid arthritis	71433	Monoarticular juvenile rheumatoid arthritis
7141	Felty’s syndrome	7144	Chronic post-rheumatic arthropathy
7142	Other rheumatoid arthritis with visceral or systemic involvement	71481	Rheumatoid lung
71430	Polyarticular juvenile rheumatoid arthritis, chronic or unspecified	71489	Other specified inflammatory polyarthropathies
71431	Polyarticular rheumatoid arthritis, acute	7149	Unspecified inflammatory polyarthropathy
71432	Pauciarticular juvenile rheumatoid arthritis	7200	Ankylosing spondylitis
CCS Code 210			
ICD-9	Dx	ICD-9	Dx
7100	Systemic lupus erythematous	7104	Polymyositis
7101	Systemic sclerosis	7108	Other specified diffuse diseases of connective tissue
7102	Sicca syndrome	7109	Unspecified diffuse diseases of connective tissue
7103	Dermatomyositis		

Variables

Primary outcomes of interest were in-hospital outcomes, including the length of stay, inpatient mortality, and total charges (Table 2). For cost analysis, the National Inpatient Sample database provides total charges that reflect the amount billed by the hospital for services rather than the actual costs of the amount received as reimbursement. Demographics including age, gender, median household income, and race were also compared for descriptive analysis. Comorbidities were established using the Elixhauser-determined comorbidities (Table 3).

**Table 2:** Comparison of primary outcomes CTDs = Connective tissue disorders; HF = Congestive heart failure Values are reported as mean with n (%) unless mentioned otherwise. Mortality, total charges, and length of stay were regressed on the group variable, which was then adjusted for demographical and comorbidity variables using generalized linear models with family binomial, gamma, and negative-binomial, respectively. A p-value smaller than 0.05 was deemed to be statistically significant.

Outcomes	HF (n=6,433,756)	HF with CTDs (n=170,721)	HF without CTDs (n=6,263,035)	p-value
Mortality	283,499 (4%)	5,884 (3.4%)	277,615 (4.4%)	<0.001
Total Charges ($)	51,731	46,256	51,880	<0.001
Length of stay (days)	6.11	5.79	6.12	<0.001

**Table 3:** The basic characteristics and comorbidities of the study population AIDS = Acquired immunodeficiency syndrome; CTDs = Connective tissue disorders; HF = Congestive heart failure Results are represented as n (%) unless mentioned otherwise. Between-group differences were tested using the chi-square test and the Wilcox rank-sum test.

	HF	HF with CTDs	HF without CTDs	p-value
Mean Age (years)	72.7	70.54	72.76	<0.001
Female Gender	3,322,774 (52%)	128,168 (75.0%)	3,194,606 (51.0%)	<0.001
		Income also(quartile)		<0.001
First quartile	2,028,499 (32%)	53,191 (32%)	1,975,308 (32%)	
Second quartile	1,662,709 (26%)	43,494 (26%)	1,619,215 (26%)	
Third quartile	1,459,119 (23%)	38,995 (23%)	1,420,124 (23%)	
Fourth quartile	1,153,846 (18%)	32,004 (19%)	1,121,842 (18%)	
		Race		<0.001
White	4,260,861 (71%)	110,379 (69%)	4,150,482 (71%)	
Black	1,040,478 (17%)	31,975 (20%)	1,008,503 (17%)	
Hispanic	405,618 (7%)	10,325 (6%)	395,293 (7%)	
Asian or Pacific Islander	102,270 (2%)	2,074 (1%)	100,196 (2%)	
Native American	32,365 (1%)	1,006 (1%)	31,359 (1%)	
Other	142,816 (2%)	3,408 (2%)	139,408 (2%)	
		Comorbidities		
AIDS	11,331 (0%)	122 (0%)	11,209 (0%)	<0.05
Alcohol Abuse	190,329 (3%)	2,315 (1%)	188,014 (3%)	<0.05
Deficiency Anemia	1904,272 (30%)	58,270 (34%)	1,846,002 (29%)	<0.05
Chronic Blood Loss Anemia	88,760 (1%)	2,350 (1%)	86,410 (1%)	0.92
Diabetes Mellitus Uncomplicated	2,069,454 (32%)	44,051 (26%)	2,025,403 (32%)	<0.05
Diabetes Mellitus Complicated	686,542 (11%)	11,784 (7%)	674,758 (11%)	<0.05
Hypertension	4,512,944 (70%)	117,664 (69%)	4,395,280 (70%)	<0.05
Hypothyroidism	1,105,917 (17%)	37,067 (22%)	1,068,850 (17%)	<0.05
Obesity	1,117138 (17%)	25,906 (15%)	1,091,232 (17%)	<0.05
Peripheral Vascular Disease	835,467 (13%)	18,269 (11%)	817,198 (13%)	<0.05
Pulmonary Circulation Disorders	425,542 (7%)	14,893 (9%)	410,649 (7%)	<0.05
Renal Failure	2,274,911 (35%)	52,224 (31%)	2,222,687 (35%)	<0.05

Statistical analysis

All variables were summarized together and separately by group, mean ± standard deviation for numerical variables, and frequency and proportion for categorical variables. The Wilcox rank-sum test or chi-square test was applied to test between-group differences. Mortality, total charges, and length of stay were regressed on the group variable, which was then adjusted for demographical and comorbidity variables using generalized linear models with family binomial, gamma, and negative-binomial, respectively. A p-value smaller than 0.05 was deemed to be statistically significant. All the statistical analyses were performed in R 3.5.5 (R Core Team, 2013, http://www.R-project.org/).

## Results

Our study included a total of 6,433,756 individuals with HF. Among these individuals, there was a total of 170,721 individuals with CTDs whom we classified as group 1 and 6,263,035 individuals without CTDs whom we classified as group 2. Primary outcomes, including inpatient mortality, length of stay, and total charges, were adjusted for demographic variables and comorbidity variables. Inpatient mortality in group 1 was lower at 3.45% as opposed to 4.43% among group 2 (p<0.001). The mean length of stay was lower in group 1 at 5.79 days as opposed to 6.12 days in group 2 (p<0.001). The mean charges were lower in group 1 at $46,256 as opposed to $51,880 in group 2 (p<0.001) (Table 2).

A comparison of basic characteristics between the two groups revealed group 1 being younger and female-dominant as shown in Table 2. There was a general trend of increased hospitalization rate with a decrease in household income for the entire cohort.

We further analyzed HF with CTDs population by stratifying patients with either systolic HF or diastolic HF (Table 4). Unfortunately, ICD coding does not differentiate heart failure based on ejection fraction but rather on whether the failure is systolic or diastolic. Diastolic HF is more prevalent and female-dominant than systolic HF in this cohort, consistent with previous findings in patients with RA [1]. Diastolic HF showed lower mortality and less mean total charges than systolic HF, after adjusting for comorbidities, including diabetes mellitus, hypertension, hypothyroidism, obesity, pulmonary circulation disorders, and renal failure. However, the length of stay was longer in patients with diastolic HF.

**Table 4:** Comparison between systolic HF and diastolic HF HF = Congestive heart failure; CTDs = Connective tissue disorders Values are reported as mean with n (%) unless mentioned otherwise. Mortality, total charges, and length of stay were regressed on the group variable, which was then adjusted for demographical and comorbidity variables using generalized linear models with family binomial, gamma, and negative-binomial, respectively. A p-value smaller than 0.05 was deemed to be statistically significant.

	Systolic HF with CTDs (n = 41,928)	Diastolic HF with CTDs (n= 66,658)	p-value
Mortality	1,466 (3.50%)	2,047 (3.07%)	<0.001
Mean charges in $	49,097	45,355	<0.001
Length of stay in days	5.84	5.96	<0.001
Age (years)	69.01	72.45	<0.001
Gender in females	27,808 (66.32%)	54,266 (81.41%)	<0.001

## Discussion

The results of our investigation showed a statistically significant decrease in mortality, length of stay, and total charges for hospitalization in HF with CTDs as compared to HF without CTDs. The difference in length of stay, though statistically significant, we realize, has little relevance clinically given the eight-hour difference. Similarly, a study done by Suero and Hajjali showed that in-hospital mortality for patients with acute myocardial infarction and CTD was lower than the general population [5]. The reason for better outcomes among hospitalized patients with a history of these inflammatory diseases is currently speculative. It is plausible that baseline anti-inflammatory treatment could have played a cardioprotective role. However, this is out of the scope of our study, as the database we used does not provide us with this information. If we had access to the medications our patients were on, it would provide great insight into their role. 

Several pro-inflammatory cytokines, including tumor necrosis factor-alpha (TNF-alpha), interferon-gamma, interleukin-1-beta, interleukin-6, interleukin-17, and interleukin-18 have been implicated in the pathogenesis of heart failure [6]. The role of TNF-alpha was recognized in 1990, and it has been known to induce cardiomyocyte dysfunction, cardiomyocyte hypertrophy, and fibrosis [6-7]. Anti-TNF-alpha targeted inhibition for cardiac dysfunction has been proven beneficial in animal models; however, this could not be translated to humans [6]. Furthermore, these medications have been shown to worsen outcomes in patients with advanced heart failure with New York Heart Association classes III-IV [7]. The mainstay of treatment for CTDs, primarily RA and SLE, which have been studied extensively, include immunomodulators like methotrexate, sulfasalazine, and hydroxychloroquine. A study done by Therneau et al. showed that methotrexate appeared to be protective against heart failure among patients with RA. It appeared to reduce the risk of developing heart failure among RA patients by half [8]. Another study done by Bernatsky et al. showed a 20% reduction in the risk of hospitalization with HF among RA patients with methotrexate [9]. Together, anti-inflammatory agents may play an important role in the disease process of heart failure.

In the HF with CTDs group, there was a significantly lower prevalence in diabetes, hypertension, renal failure, peripheral vascular disease, obesity, and alcohol abuse. These comorbidities are known to be independent risk factors for HF. Nevertheless, in-hospital outcomes remain statistically different after adjusting for the aforementioned comorbidities.

Analysis of socioeconomic trends revealed a significantly higher rate of hospitalization among patients with a lower household income. This was in agreement with the findings previously seen in multiple studies [10] and again highlighted the socioeconomic disparities in health.

Further examination of systolic HF and diastolic HF subgroups has shown a similar trend in inpatient outcomes, including lower in-hospital mortality and mean charges in the diastolic HF subgroup. Our results are in line with the general trends observed in the entire HF population, despite the much higher female prevalence in this CTDs subpopulation.

Limitations

The national inpatient sample data represents hospitalization episodes and does not contain information about individual patient’s medications or rehospitalization. Moreover, coding inaccuracies and inconsistencies are inevitable. Despite using CCS codes to identify clinical conditions, it is based on ICD codes, and relying on these ICD codes results in the exclusion of about one-third of the patients with clinical evidence of HF. This is further worsened as data is usually analyzed by the first listed diagnosis. Cost estimates used based on this data are amounts billed by the hospital and not insurance reimbursement rates. Despite using Elixhauser comorbidities in our study, these have been developed for use in administrative rather than clinical studies. Further data points differentiating index diagnosis admissions and readmission for HF would have further added strength to the study. A new diagnosis of HF is usually worked up intensively with procedures such as left heart catheterization, for instance, and can add to the overall cost of the admission. Also, due to the limitations of the database, we were unable to differentiate between left heart failure and right heart failure, which would add another dimension to the study.

## Conclusions

To our knowledge, this is the first study analyzing HF inpatient outcomes related to underlying CTDs. Overall, the outcomes in HF patients with CTDs were better when compared with HF patients without CTDs. Further research with a prospective design will help validate the findings and provide insight into the mechanisms.
